# Dasatinib synergizes with JSI-124 to inhibit growth and migration and induce apoptosis of malignant human glioma cells

**DOI:** 10.4103/1477-3163.65448

**Published:** 2010-07-14

**Authors:** Daniel R. Premkumar, Esther P. Jane, Naomi R. Agostino, Joseph L. Scialabba, Ian F. Pollack

**Affiliations:** 1 Department of Neurosurgery, University of Pittsburgh Medical Center.; 2 Department of University of Pittsburgh School of Medicine, University of Pittsburgh Medical Center; 3 Department of University of Pittsburgh Cancer Institute Brain Tumor Center, University of Pittsburgh Medical Center

**Keywords:** Apoptosis, dasatinib, glioma, JSI-124, migration

## Abstract

**Background:**

Src family kinases (SFK) collectively regulate a variety of cellular functions in many cancer types, including proliferation, invasion, motility, survival, differentiation, and angiogenesis. Although Dasatinib (BMS-354825), an ATP-competitive, small molecule tyrosine kinase inhibitor, suppresses the activity of SFKs at nanomolar concentrations, IC50 values for antiproliferative effects in glioma cell lines were well above the clinically achievable range, suggesting the need to interfere with other components of receptor-induced downstream signaling in order to achieve an optimal therapeutic effect.

**Materials and Methods:**

The cytotoxic effects of combining Src and STAT3 inhibition on glioma cell lines were evaluated using assays to measure cell proliferation, apoptosis and migration. Western blotting and immunocytochemistry was used to monitor its effects on cell signaling and morphology.

**Results:**

Silencing Src and STAT3 expression each partially inhibited cell proliferation and migration. In addition, JSI-124 significantly enhanced the efficacy of dasatinib in vitro. Combination of dasatinib and JSI-124 achieved significant inhibition of migration in all cell lines, which correlated with the inhibition of Src and downstream mediators of adhesion (e.g. focal adhesion kinase). Cells exposed to dasatinib and JSI-124 exhibited morphological changes that were consistent with an upstream role for Src in regulating focal adhesion complexes.

**Conclusions:**

Targeting the Src and STAT pathways may contribute to the treatment of cancers that demonstrate increased levels of these signaling mediators, including malignant human glioma. Clinical studies in these tumor types are warranted.

## INTRODUCTION

Malignant gliomas are biologically aggressive tumors that have proven largely refractory to conventional treatment modalities, such as surgery, irradiation, and cytotoxic chemotherapy.[1,2] These tumors exhibit a host of molecular alterations that play a role in their aberrant proliferation and invasiveness, including constitutive activation of several cell surface tyrosine kinase receptors, such as EGFR and PDGFR. These alterations activate critical downstream mediators that contribute to the neoplastic phenotype, and may constitute promising therapeutic targets. However, the complexity of the interactions between cell surface receptors and downstream signaling targets has called into question the clinical utility of blocking any target in isolation, and the results of single-agent-based strategies have to date been disappointing.

In that regard, Src family members are known to play an important role in mediating the proliferative and invasive effects of EGFR activation,[3–6] although a parallel pathway of EGFR signaling occurs via STATs (signal transducers and activators of transcription),[7] which undergo phosphorylation, dimerization, and nuclear translocation to regulate gene targets.[8,9] Src kinases themselves may activate STATs,[10–13] and, in this context, Src family members can serve both as intermediates between tumorigenic tyrosine kinases and STAT activation [14] and as direct mediators of various aspects of the neoplastic phenotype.[15,16]

Dasatinib is an orally available Src family kinase inhibitor [17] that inhibits Abl and SFKs at low nanomolar concentrations. At higher concentrations, dasatinib inhibits other tyrosine kinases such as Akt, FAK, and the receptor tyrosine kinases PDGFR, c-kit, and Ephrin.[18] Dasatinib has been shown to inhibit c-Src-mediated downstream pathways in a variety of malignant cell lines such as prostate, head and neck, and lung cancer cells,[19–22] as well as in glioma cells.[6] However, single-agent antiproliferative activity in glioma and other solid tumor models has often required concentrations above the clinically achievable range,[23–26] suggesting the need to interfere with other components of receptor-induced downstream signaling to achieve an optimal therapeutic effect.

Given the interactions between receptor tyrosine kinases and both Src family and STAT activation, we hypothesized that combining Src with STAT inhibition might achieve improved efficacy in glioma cell lines. To date, a variety of strategies have been used to inhibit activated STAT3 for cancer treatment and independent activity of several of these has been observed in gliomas.[27–29] We have previously shown that AG490 inhibited the growth of glioma cell lines [30] and that JSI-124 (cucurbitacin I), a cell-permeable triterpenoid inhibitor of JAK/STAT3,[31,32] had antiproliferative and immunopotentiating effects.[33] Studies from other laboratories have shown that JSI-124 inhibits proliferation and viability of EGFR - and EGFRvIII-expressing glioma cell lines.[32] We therefore examined the potential for STAT inhibition with JSI-124 to enhance the efficacy of Src inhibition with dasatinib in glioma cells. We found that although each agent had a moderate level of independent efficacy, the combination of JSI-124 and dasatinib synergistically decreased cell proliferation and viability and had significant effects on cell migration that far exceeded those observed with either drug alone.

## MATERIALS AND METHODS

### Inhibitors and reagents

Dasatinib and JSI-124 were purchased from Chemie Tek (Indianapolis, IN) and Calbiochem (Cambridge, MA), respectively. Stock solutions were prepared in DMSO and stored at -20° C. Unless otherwise mentioned, all antibodies used for this study were purchased from Cell Signaling Technology, Inc. (Beverly, MA).

### Cell culture

The established malignant glioma cell lines U87, T98G, U373, LN18, LN229 and A172 were obtained from the American Type Culture Collection (Manassas, VA). LNZ308 and LNZ428 were provided by Dr. Nicolas de Tribolet. U87, T98G, and U373 were grown in minimum essential medium (MEM) supplemented with sodium pyruvate and nonessential amino acids; LN18, LN229, A172, LNZ308, and LNZ428 were cultured in α-MEM supplemented with L-glutamine. Human astrocytes (HA) and human cerebellar astrocytes (HAC) were obtained from ScienCell Research Laboratories (San Diego, CA) and cultured in Astrocyte Growth Medium. All growth media contained 10% fetal calf serum, L-glutamine, non-essential amino acids and sodium pyruvate, 100 IU/ml penicillin, 100 mg/ml streptomycin, and 0.25 mg/ml amphotericin (Invitrogen, Carlsbad, CA).

### Cell proliferation and cytotoxicity assays

Cells (5 × 0^3^/well) were plated in 96 -well microtiter plates (Costar, Cambridge, MA) in 100 *µ*l of growth medium, and after overnight attachment, were exposed for three days to a range of concentrations of JSI-124 and dasatinib, alone and in combination. Control cells received vehicle alone (DMSO). Cells were then washed in inhibitor-free medium, and viable cell numbers were determined using a colorimetric assay (CellTiter96 Aqueous Non-Radioactive Cell Proliferation Assay; Promega, Madison, WI) as per the supplier’s protocol.[34] All studies were conducted in triplicate and repeated at least three times.

To assess tumor cytotoxicity, 2.5 × 10^5^ cells were seeded in six-well dishes and after overnight attachment, were treated with selected concentrations of inhibitors or vehicle. Cells were harvested, stained with trypan blue, and counted using a hemacytometer. All samples were tested in triplicate. Viable (trypan blue-excluding) and dead cell numbers were plotted as a function of inhibitor concentration.

### Clonogenic growth assay

The effect of different inhibitor concentrations on cell viability was also assessed using a clonogenic assay. For this analysis, 250 cells were plated in six-well trays in growth medium, and after overnight attachment, cells were exposed to selected inhibitor concentrations or vehicle for one day. Cells were then washed with inhibitor-free medium and allowed to grow for two weeks under inhibitor-free conditions. Colonies were then counted. All studies were performed in triplicate.

### Immunoprecipitation and Western blotting analysis

Treated and untreated cells were washed in cold PBS and lysed in buffer containing 30 mM HEPES, 10% glycerol, 1% Triton X-100, 100 mM NaCl, 10 mM MgCl_2_, 5 mM EDTA, 2mM Na_3_VO_4_, 2 mM β-glycerophosphate, 1 mM phenylmethylsulfonyl fluoride, 1 mM 4-(2 -aminoethyl) benzenesulfonyl fluoride, 0.8 *µ*M aprotinin, 50 *µ*M bestatin, 15 *µ*M E- 64, 20 *µ*M leupeptin, and 10 *µ*M pepstatin A for 15 min on ice. Samples were centrifuged at 12,000 *g* for 15 min, supernatants were isolated, and protein was quantified using Protein Assay Reagent (Pierce Chemical, Rockford, IL). Equal amounts of protein were separated by SDS polyacrylamide gel electrophoresis (PAGE) and electrotransferred onto a nylon membrane (Invitrogen). Nonspecific antibody binding was blocked by incubation of the blots with 2% bovine serum albumin in Tris-buffered saline (TBS)/Tween 20 (0.1%) for 1 h at room temperature. The blots were then probed with appropriate dilutions of primary antibody overnight at 4°C. The antibody-labeled blots were washed three times in TBS/Tween 20 for 15 min and then incubated with a 1:1500 dilution of horseradish peroxidase-conjugated secondary antibody (Santa Cruz Biotechnology, Inc.) in TBS/Tween 20 at room temperature for 1 h. After additional washing in TBS/Tween 20, the proteins were visualized by Western Blot Chemiluminescence Reagent (Cell Signaling Technology Inc., Beverly, MA). Where indicated, the blots were reprobed with antibodies against β-actin (Sigma-Aldrich, St. Louis, MO) to ensure equal loading and transfer of proteins.

For immunoprecipitation, cells were harvested in lysis buffer. Lysates were cleared of insoluble material by centrifugation at 12,000 × *g* for 15 min at 4 °C. Equal amounts of protein (300 *µ*g) were incubated with 3 to 4 *µ*g of indicated antibodies overnight at 4 °C and protein G-conjugated beads for another 3 h. Beads were washed three times with cell lysis buffer, and proteins eluted with SDS sample buffer for Western blotting analysis as described above. Scanning densitometry was performed on Western blots using acquisition into Adobe Photoshop (Adobe Systems, Inc., San Jose, CA) followed by image analysis (UN-SCAN-IT gel™, version 6.1, Silk Scientific, Orem, UT).

### Annexin V apoptosis assay

Apoptosis induction in control (DMSO-treated) or inhibitor-treated cells was assayed by the detection of membrane externalization of phosphatidylserine with Annexin V-FITC conjugate using an Annexin V assay kit according to the manufacturer’s protocol (Molecular Probes). In brief, 2 × 10^5^ cells were harvested at various intervals after treatment and washed twice with ice-cold phosphate-buffered saline (PBS) and resuspended in 200 *µ*l of binding buffer. Annexin V-FITC and 1 *µ*g/ml propidium iodide (PI) were added and cells were incubated for 15 min in a dark environment. The reaction was stopped by adding 300 *µ*l of 1 × binding buffer, and labeling was analyzed by flow cytometry with a FACSCalibur flow cytometer (BD Biosciences, San Jose, CA).

### Transwell migration assay

Transwell inserts (Corning Life Sciences, Acton, MA) with 8-*µ*m pore size, precoated with collagen, were used to assess glioma cell migration. Cells were seeded at 60% confluence and allowed to attach for 18 h. Cells were then serum-starved for 12 h and trypsinized and 200 *µ*l of cell suspension (5 × 10^5^ cells/ml) containing different concentrations of inhibitors were added in triplicate to the upper wells. Serum-containing medium was added to the lower wells as a chemoattractant. Cells were incubated at 37°C for 16 h. Non-invasive cells on the upper surface were removed with cotton swabs, and invaded cells on the lower surface were fixed in 4% paraformaldehyde and stained with Diff-Quik II solution (Dade Behring, Marburgh, Germany). Migrated cells were counted by light microscopy, using six randomly chosen fields per filter. Statistical significance between untreated and inhibitor-treated cells was calculated with Students *t*-test. Differences were considered significant at *P* values <0.05.

### Transfection with small interfering RNA

All small interfering RNA molecules (siRNAs) were predesigned (ON-TARGET*plus* SMARTpool) by Dharmacon and transfected according to the manufacturer’s protocol. In brief, cells in 96-well plates (for cell proliferation assay) or six-well plates (for transwell migration assay) were grown to 70% confluency and transfected with siRNA for Src or STAT or a combination of both using DharmaFECT siRNA transfection reagent. Controls included cells that were mock transfected (i.e. no siRNA) and those transfected with a nontargeting (scrambled) siRNA. Cells were incubated under these conditions for 72 h. Cell proliferation and migration was measured essentially as described above.

### Immunofluorescence and assessment of apoptotic events

Cells were plated in chamber culture slides (BD Falcon, Bedford, MA). After overnight incubation, cells were fixed in phosphate-buffered saline (PBS) containing 4% formaldehyde, permeabilized with 0.1% Triton X-100 in PBS for 10 min, and incubated in blocking buffer (PBS containing 1% goat serum, 0.3% bovine serum albumin (BSA) and 0.2% Triton X-100) for 60 min. Cells were washed and incubated with anti-cytochrome *c* antibody (1:100, clone 6H2.B4; PharMingen) and anti-apoptosis-inducing factor (1:50, Santa Cruz) overnight at 4° C. Following PBS wash, cells were incubated with secondary antibody (goat anti-mouse Alexa 555 and goat anti-rabbit Alexa 488 and Hoechst 33258 (Invitrogen) for 60 min. Cells were then rinsed three times with PBS, and coverslips were mounted onto slides.

Morphological changes, such as cell shrinkage, rounding, and membrane blebbing, were evaluated by microscopic inspection of cells. In brief, cells were fixed (4% formaldehyde), permeabilized (PBS containing 0.2% Triton X-100 for 10 min), and stained for filamentous actin using Alex Fluor 488-conjugated phalloidin (diluted 1:100 in PBS, for 60 min at room temperature) and Hoechst 33258. After rinsing in PBS containing 0.2% Triton X-100, the slides were preserved at 4°C until imaging. Images were taken using an Olympus FluoView 1000 confocal microscope. Images were assembled using Adobe Photoshop CS2 software (Adobe Systems).

### Analysis of combinatorial effects

The significance of differences between experimental conditions was determined using a two-tailed Student’s t test. MTS assays were used to determine inhibition of cell survival after a 72-h treatment of multiple cell lines with different ratios of dasatinib and JSI-124. IC_50_ concentrations and combination indices for dasatinib and JSI-124 were calculated using a commercially available software program (Calcusyn; Biosoft, Ferguson, MO).[35]

## RESULTS

### Dasatinib inhibits Src activity and downstream signaling

Dasatinib has been reported to inhibit Src family kinases (SFKs).[20,21] To characterize SFK inhibition, we examined the effect of dasatinib in a panel of glioma cell lines. Dasatinib caused complete inhibition of Src activity, as measured by phosphorylation at Y416 after treatment for 24 h with concentrations of 50 nM or higher [Figure 1a]. Cells treated with dasatinib (100 nM) demonstrated inhibition of Src phosphorylation within 3 h of incubation, although total Src levels were not affected [Figure 1b]. To examine the effects of dasatinib on known downstream targets of Src, U87 cells were treated with either vehicle or varying concentrations [Figure 1c] or durations [Figure 1d] of dasatinib, and the cell lysates were immunoprecipitated with antibodies against Src family members and processed for Western blotting with antiphosphotyrosine antibody (PY20) to assess phosphorylation status. FAK phosphorylation was decreased both in a dose - and time-dependent manner [Figure 1c and Figure 1d upper panel]. Complete inhibition of Lck [Figure 1c and Figure 1d middle panel] and modest inhibition of Lyn [Figure 1c and Figure 1d lower panel] phosphorylation was also observed.

**Figure 1a-d:**
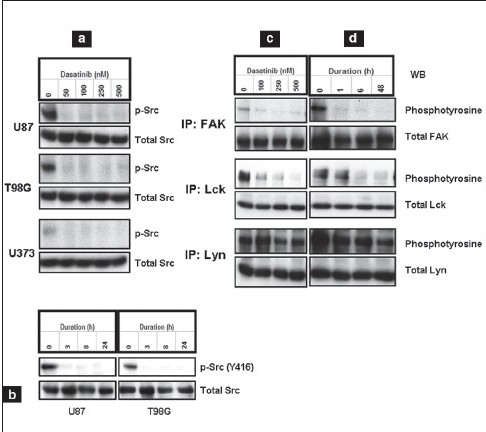
Dasatinib inhibits Src phosphorylation and downstream signaling. Cells were treated with varying concentrations of dasatinib for 1 day (a) or with 100 nM dasatinib for various durations (b). Equal amounts of protein were separated and subjected to Western blotting analysis with the indicated antibodies. U87 cells were treated with varying concentrations of dasatinib for 1 day (c) or treated with 100 nM dasatinib for the indicated durations (e). FAK, Lck and Lyn were immunoprecipitated (IP) and immunoblotted (WB) with the indicated antibodies as described in Materials and Methods

Because c-Src can affect cellular proliferation and survival by activating STAT,[36] we examined the effect of dasatinib on STAT phosphorylation in three established glioma cell lines [Figure 1e]. No inhibition of STAT3 phosphorylation was observed [Figure 1e]. Because Src can positively regulate Akt through PI3K, and the tumor suppressor PTEN (phosphatase and tensin homologue) negatively regulates the PI3K/Akt signaling pathway,[37] we examined the expression and phosphorylation of Akt in LN18 (PTEN wild type), U373 (PTEN mutant), and U87 and LNZ308 (PTEN deleted) cell lines. Consistent with the characteristics of many tumors, glioma cells exhibited a high level of basal Akt activity. Minimal or no inhibition was observed in Akt phosphorylation (S473) in response to dasatinib, irrespective of PTEN status [Figure 1f, upper panel], even at a concentration of 500 nM [Figure 1f, lower panel]. In addition, no inhibition of ERK 1/2 phosphorylation was observed [Figure 1g].

**Figure 1e-g:**
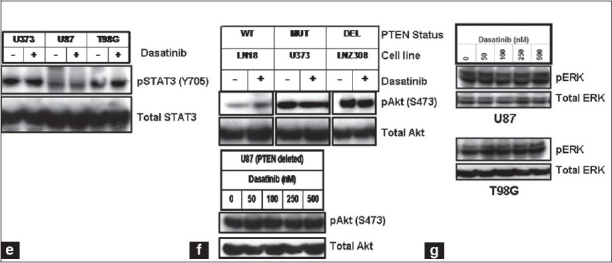
Dasatinib inhibits Src phosphorylation and downstream signaling. (e) U373, U87, and T98G cells were treated with dasatinib (100 nM) for 1 day. (f) LN18, U373, and LNZ308 cells were treated with dasatinib (100 nM; upper panel) or U87 cells with varying concentrations of dasatinib (lower panel) for 1 day. (g) U87 and T98G cells were treated with varying concentrations of dasatinib for 1 day. Equal amounts of protein were separated and Western blot analysis was performed with the indicated antibodies

### Dasatinib treatment inhibits cell proliferation and cell migration and disrupts cytoskeletal organization

To examine the effects of dasatinib on glioma cell viability, U87 and U373 cells were treated with a range of concentrations of dasatinib in complete medium for 48 h, stained with both propidium iodide and Annexin V, and analyzed by fluorescence-activated cell sorting analysis. No change in cell viability was observed at 100 nM dasatinib, whereas modest levels of apoptosis were detected at high concentrations (500 nM, Figure 2a. We then examined the effect of dasatinib on cell proliferation of a panel of glioma cell lines. Eight established glioma cell lines were cultured with increasing concentrations of dasatinib for three days and cell proliferation was assessed by MTS assay. Dasatinib inhibited cell proliferation in a dose-dependent manner [Figure 2b], although the IC50 levels ranged between 7 and 50 *µ*M [Figure 2b], significantly higher than the achievable concentrations of dasatinib in clinical trials (100 nM).[38–41]

**Figure 2a-b:**
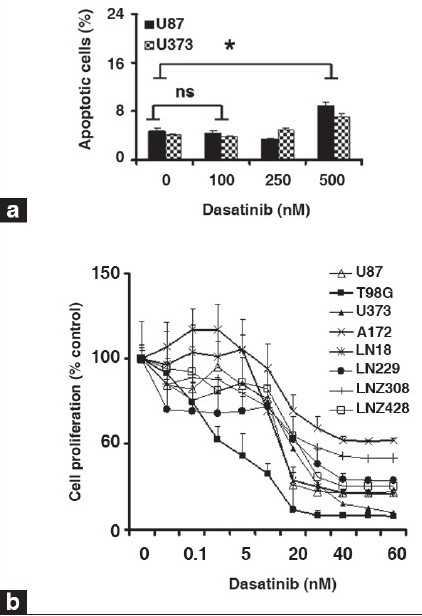
Dasatinib treatment inhibits cell proliferation and migration and disrupts cytoskeletal organization. (a). U87 and U373 cells were treated with varying concentrations of dasatinib for 2 days. FACS analysis was performed as described in Materials and Methods. The values represent the mean ± standard deviation for two separate experiments performed in triplicate (ns = not significant; *, p < 0.05 versus control). (b) The relationship between dasatinib and cell numbers was assessed by spectrophotometric measurement of MTS bioreduction as described in the Materials and Methods

Despite the limited effects of dasatinib on glioma cell proliferation and viability, significant inhibition of glioma cell migration was observed using a Transwell migration assay.[42] Whereas untreated U87, T98G, A172, LN18, LN229 and U373 cells were highly motile [Figure 2c, upper panel], dasatinib treatment (100 nM) substantially decreased migration [Figure 2c, lower panel]. Migration through the filter was decreased by 88, 76, 67, 44, 57 and 94% in U87, T98G, LN18, A172, LN229 and U373, respectively, with 100 nM dasatinib treatments [Figure 2d]. Moreover, following dasatinib treatment, cells displayed morphological alterations, adopting a round, contracted appearance. As shown in Figure 2e, the cytoskeletal structure of the cells was disrupted, creating a dense and compact cell body in which the intricate actin-branching structures appeared to have collapsed. Taken together, dasatinib resulted in significant alterations in cell migration but did not inhibit cell growth at physiologically relevant concentrations.

**Figure 2c-d:**
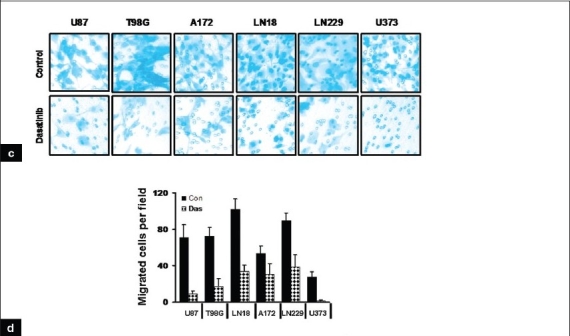
Dasatinib treatment inhibits cell proliferation and migration and disrupts cytoskeletal organization. (c). Transwell migration analysis was performed with or without dasatinib (100 nM) as described in the Materials and Methods. At the end of the experiment, cells on the lower part of the membrane was fixed, stained, and images were taken. (d) Migrated cells were counted as described in the Materials and Methods. Glioma cells incubated with dasatinib (100 nM) showed 40-90% reduction in migration through pores compared with untreated control cells

**Figure 2e:**
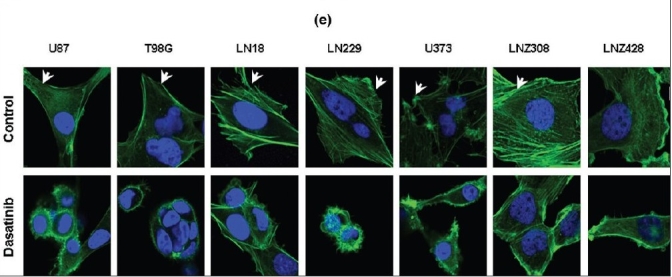
Dasatinib treatment inhibits cell proliferation and migration and disrupts cytoskeletal organization. (e) Seven glioma cell lines were seeded at 60% confl uence and allowed to attach overnight. On the following day, cells were treated with or without dasatinib (100 nM) for 1 day. Cells were fixed, permeabilized, and stained with Hoechst 33342 and Alexa Flour 488 phalloidin to visualize nuclear and cellular morphology. Untreated control cells develop broad lamellipodia (arrows). Dasatinib causes morphological changes such as cell shrinkage, rounding, and membrane blebbing

### Inhibition of Src and STAT3 signaling suppresses glioma cell growth and migration

**Figure 3a-c:**
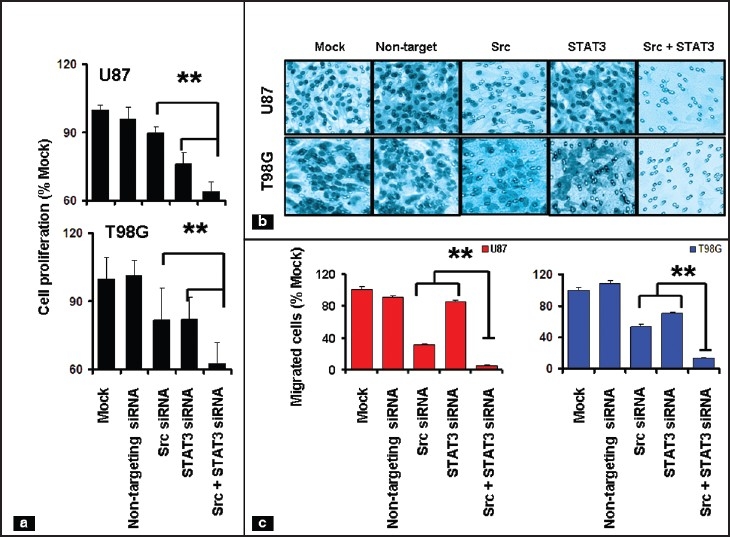
Inhibition of Src and STAT3 signaling suppresses glioma cell growth and migration. (a) The relationship between specific siRNA or mock transfection and cell numbers was assessed semiquantitatively by spectrophotometric measurement of MTS bioreduction. (b) U87 and T98G cells were transfected with indicated siRNA. Cell migration assay was performed as described in Materials and Methods and representative images were shown. (c) Migrated cells were presented as a percentage of mock-transfected control. **P< 0.05, combination of Src and STAT siRNA versus individual siRNA

Because STAT3 has been implicated in malignant transformation and tumor cell survival,[29,43–45] and in interacting with Src-dependent signaling, we examined whether STAT3 knockdown would enhance the effects of Src inhibition in glioma cell lines. Accordingly, we transfected U87 and T98G cells with STAT3 siRNA, c-Src siRNA, the combination of both c-Src and STAT3 siRNA, or non-targeting siRNA, and subsequently measured cell proliferation. Mock transfected cells received transfection reagents without siRNA. As shown in Figure 3a, depletion of both c-Src and STAT3 resulted in modest inhibition of cell proliferation, whereas following combination of c-Src and STAT3 siRNA treatment, cell proliferation was decreased by 40%.

We also assessed the functional role of c-Src and STAT3 in glioma cell migration by examining the effects of knockdown of Src, STAT3 or the combination of both by siRNA [Figure 3b]. Although transfection with 50 nM of c-Src and STAT3 siRNA each decreased cell migration to some extent, the combination produced nearly complete abrogation of migratory function. The number of U87 cells able to migrate through the filter decreased by 70 and 15.4% upon treatment with Src and STAT3 siRNA, respectively, when compared to cells transfected with non-specific siRNA. In T98G cells, migration was 47 and 30% lower in cells transfected with Src and STAT3 siRNA, respectively. However, combined inhibition of both c-Src and STAT3 almost completely abolished cell migration [Figure 3b and Figure 3c]. These data show that c-Src and STAT3 cooperate to maintain cell survival, proliferation and migration in glioma cell lines.

### JSI-124 inhibits STAT3 and downstream signaling

Because JSI-124 has been shown to pharmacologically inhibit STAT3 phosphorylation,[31] we hypothesized that the combination of dasatinib and JSI-124 might cooperate to block glioma cell proliferation and induce apoptosis. To determine whether JSI-124 suppresses phosphotyrosine STAT3 levels in human glioma cell lines, cell lysates were processed for Western blotting with antiphosphotyrosine STAT3. STAT3 phosphorylation was inhibited by JSI-124 [Figure 4a, upper panel], whereas no effect on total STAT3 levels was observed [Figure 4a, lower panel]. Then we evaluated the effects of JSI-124 on the phosphotyrosine levels of JAK1, JAK2, and Src. Figure 4b shows that treatment of glioma cells with JSI-124 results in no reduction of tyrosine phosphorylated JAK1 and JAK2, and minimal reduction in the levels of tyrosine-phosphorylated Src. In contrast, STAT3 inhibition with JSI-124 reduced the expression of several known STAT3 downstream genes, such as Bcl2, cyclin E and survivin, as shown in Figure 4c. We further examined the expression of proteins involved in cell cycle regulation and apoptotic pathways, which are known to be regulated by the JAK/STAT pathway. The cyclin kinase inhibitor p21WAF1, which is associated with cell cycle arrest and apoptosis, was increased in a p53 independent manner [Figure 4c].

**Figure 4a-c:**
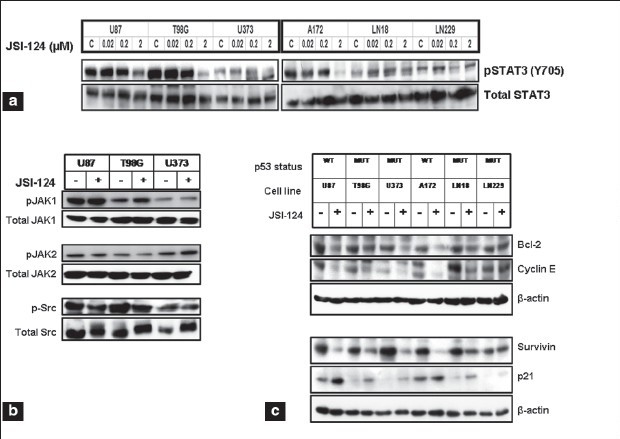
JSI-124 inhibits STAT and downstream signaling. (a) Glioma cells were treated with indicated concentrations of JSI-124 for 1 day and Western blot analysis was performed with pSTAT3 (Y705) antibody and reprobed with total STAT3 antibody. (b) U87, T98G and U373 cells were treated with or without JSI-124 (1 µM) for 1 day and cell lysates were subjected to Western blot analysis with indicated antibodies. (c) Cells were treated with or without JSI-124 (1 µM) for 1 day and cell lysates were subjected to Western blot analysis with indicated antibodies. β-actin served as loading control

We then examined the effect of JSI-124 on cell proliferation and clonogenicity in glioma cell lines. JSI-124 inhibited both cell proliferation [Figure 4d] and clonogenicity [Figure 4e] in a dose-dependent manner. In order to confirm the specificity of the inhibitor toward tumor cells, we compared the effect of JSI-124 on normal cells (HA, HAC). As shown in Figure 4f, exposure to high concentrations of JSI-124 (4 *µ*M) had minimal toxicity in non-neoplastic astrocytes, demonstrating the selectivity against tumor cells.

**Figure 4d-f:**
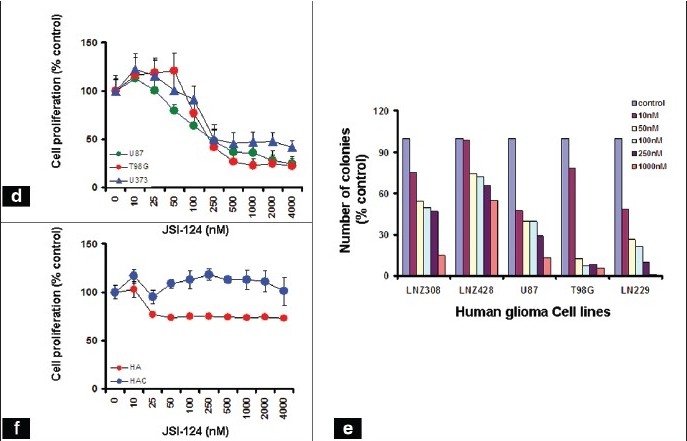
JSI-124 inhibits STAT and downstream signaling. (d) The relationship between JSI-124 and glioma cell growth was assessed MTS bioreduction. (e) Graph showing the relationship between colony counts and concentration of JSI-124 was done as described in Materials and Methods. (f) The relationship between JSI-124 concentration and proliferation of human astrocytes (HA) and human cerebellar astrocytes (HAC) was assessed by MTS assay. Although there was a dose-dependent reduction of cell growth in the glioma cell lines, minimal (HA) or no (HAC) reduction of cell numbers was observed in non-neoplastic astrocyte cultures

### Cotreatment with dasatinib and JSI-124 synergistically inhibits growth and induces apoptosis

Because the concentrations of dasatinib required to inhibit cell proliferation and induce apoptosis were noted to be above the clinically achievable range, we questioned whether the activity of this agent could be enhanced by combination with STAT3 pathway inhibition. To characterize the potential interactions between dasatinib and JSI-124 on cell viability, human glioma cell lines were exposed to dasatinib or JSI-124 or the combination of both and cell viability was assessed after three days. As shown in Figure 5a, the combination of both inhibitors was substantially more effective than either single agent and showed a significant decrease in cell numbers.

To determine whether the enhancement of glioma cell cytotoxicity by the combination of dasatinib and JSI-124 reflected additive or synergistic interactions, we performed concentration-effect and isobologram analyses. The data were then applied to determine the combination index (CI), which provides a semiquantitative assessment of the presence of additive, synergistic, or antagonistic interactions at different effect levels.[35] CI is 1 for additive interactions, greater than 1 for antagonistic interactions, and less than 1 for synergistic interactions. The combination of dasatinib and JSI-124 produced a synergistic inhibition, based on the observation that the CI was substantially less than 1 in all glioma cell lines analyzed [Figure 5b].

**Figure 5a-b:**
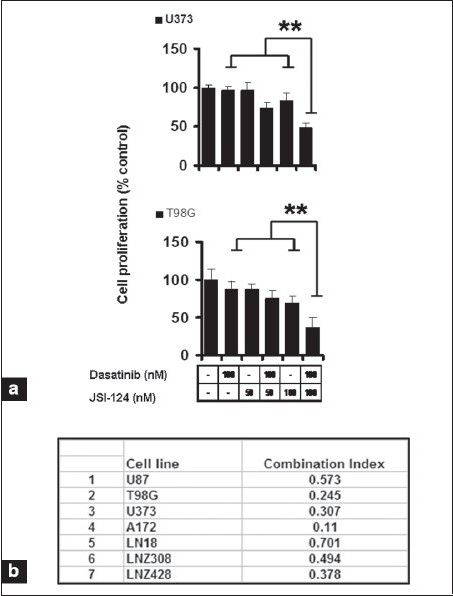
Cotreatment with dasatinib and JSI-124 synergistically inhibits growth and induces apoptosis of glioma cells. (a) The relationship between JSI-124 and dasatinib concentration and cell numbers was assessed by MTS assay. *P* < 0.005 combination of dasatinib and JSI-124 versus single agent alone. (b) Glioma cells were exposed to varying concentrations of dasatinib and JSI-124 at a fixed molar ratio (1: 2) for 3 days. MTS assay was performed and the data were then used to calculate the combination index (CI) using commercially available software (Calcusyn; Biosoft).

In order to determine whether this synergistic effect was specific against glioma cells compared to non-neoplastic astrocytes, we also examined the effect of JSI-124 alone or in combination with dasatinib on normal cells (HA, HAC). Cells were treated with 100 nM dasatinib or indicated concentrations of JSI-124 or the combination of both for three days, and cell proliferation was assessed by MTS assay. As shown in Figure 5c, the combination of dasatinib and JSI-124 had little or no effect on normal cells with minimal reduction from control in human astrocytes (HA), and human cerebellar astrocytes (HAC).

We also examined whether the synergistic efficacy of JSI-124 and dasatinib reflected the induction of apoptosis. U87 cells treated with these agents alone or in combination for 48 h were stained with annexin V and PI and analyzed by flow cytometry. Annexin V binds to phosphatidylserine, which translocates from the inner leaflet to the outer leaflet of the plasma membrane in apoptotic cells. Annexin V staining thus provides a marker for cells that are undergoing apoptosis. PI staining provides a measure of cell viability and is used to distinguish between cells in early and late apoptosis. Greater than 90% of U87 cells treated with DMSO (control) were negative for both PI and Annexin V (*lower left quadrant*) and thus viable [Figure 5d]. Similar profiles were observed for cells treated with dasatinib or JSI-124 alone. However, there was a large decrease in viable cells after treatment with the combination of both inhibitors.

**Figure 5c-d:**
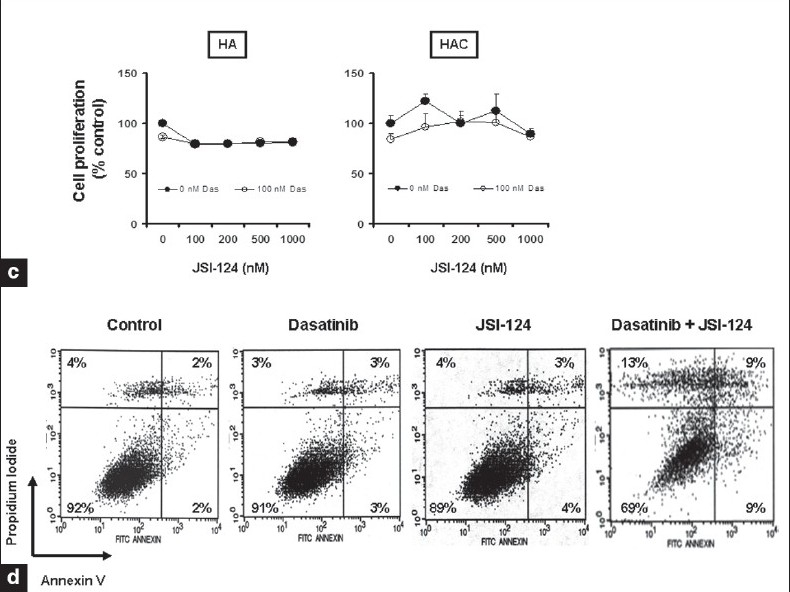
Cotreatment with dasatinib and JSI-124 synergistically inhibits growth and induces apoptosis of glioma cells. (c). The relationship between JSI-124 and dasatinib concentration and astrocyte cell numbers was assessed by MTS assay. (d) U87 cells were treated with dasatinib (100 nM), JSI-124 (200 nM) or the combination of both for 2 days. FACS analysis was performed as described in Materials and Methods

### Cytochrome *c* and apoptosis-inducing factor (AIF) release is an early event of dasatinib and JSI-124-induced apoptosis

Redistribution of cytochrome *c* and AIF has been reported to be an early event of the apoptotic process.[46–48] To evaluate the enhancement of glioma cell cytotoxicity by the combination of dasatinib and JSI-124, we examined the involvement of cytochrome *c* and AIF by immunofluorescence microscopy. Immunofluorescence detection of AIF and cytochrome *c* in untreated control cells normally yields a punctate cytoplasmic staining pattern with some preference for the perinuclear area [Figure 6a]. This staining profile is typical for mitochondrial localization.[49] Dual staining experiments, which allow for the simultaneous detection of cytochrome *c*, indicate a coincidence between AIF and cytochrome *c*. Cells incubated (1 h) with the combination of dasatinib and JSI-124 showed increased diffuse staining of cytochrome *c* in the cytoplasm and translocation of AIF from the mitochondria into the nucleus [Figure 6a]. Co-treated cells also displayed morphological changes, characterized by a round, contracted appearance. As seen in Figure 6b, the actin cytoskeletal structure of the cells was disrupted, creating a dense and compact cell body in which the intricate actin-branching structures appeared to have collapsed and filamentous tubulin was compacted.

**Figure 6a:**
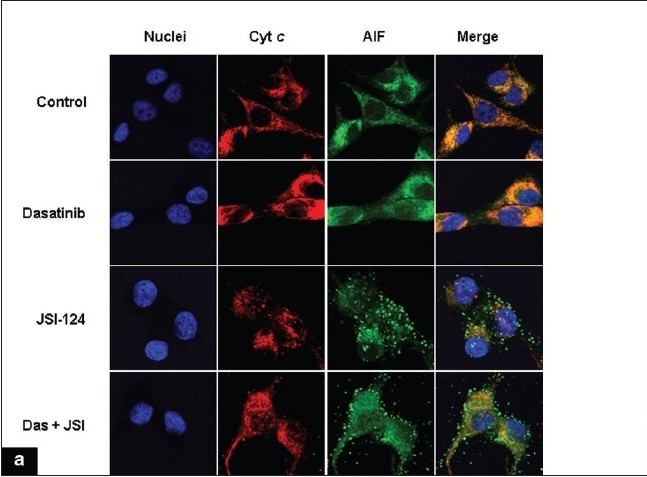
Release of cytochrome c and apoptosis inducing factor (AIF) is associated with apoptosis induced by dasatinib and JSI-124 treatment. (a) U87 cells were seeded at 60% confl uence and allowed to attach overnight. Cells were incubated with dasatinib (100 nM) or JSI-124 (200 nM) or the combination of both for 1 h. Cells were then fixed, permeabilized, and stained with antibodies specific for cytochrome c and AIF as described under Materials and Methods, and counterstained with Hoechst 33342 to visualize nuclei. This experiment was repeated three times, yielding comparable results

We also examined the effect of these inhibitors on other biochemical markers of apoptotic signaling in U87 cells. As shown in Figure 6c, treatment with dasatinib and JSI-124 alone had little effect on the levels of cleaved Bax, caspase 3, or PARP. In contrast, combined treatment with both agents resulted in a significant increase in the cleaved forms of Bax, caspase 3 and PARP. Conversely, following dasatinib and JSI-124 treatment, survivin levels were decreased by 55%.

**Figure 6b-c:**
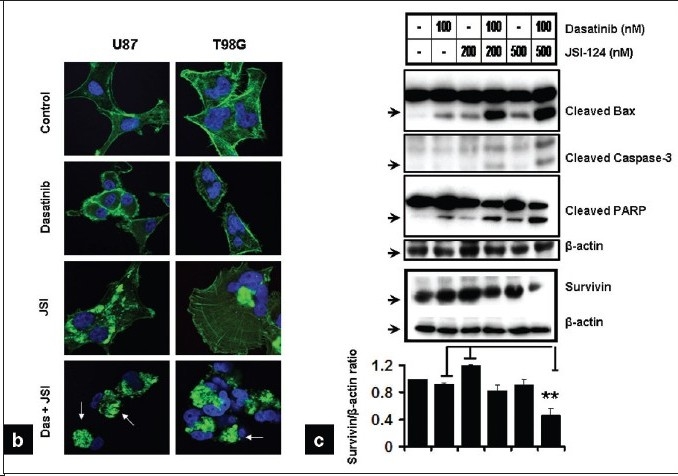
Release of Cytochrome c and apoptosis inducing factor (AIF) is associated with apoptosis induced by dasatinib and JSI-124 treatment. (b) U87 and T98G cells were treated as shown for 1 day. Combination of dasatinib and JSI-124 causes morphological changes such as cell shrinkage, rounding, membrane blebbing and nuclear condensation (arrows). (c) U87 cells were treated with inhibitors for 2 days and Western blotting and densitometric analysis was performed as described in the Materials and Methods

### Effect of dasatinib and JSI-124-induced apoptosis on downstream growth signaling pathways in glioma cells and non-neoplastic astrocytes

**Figure 7:**
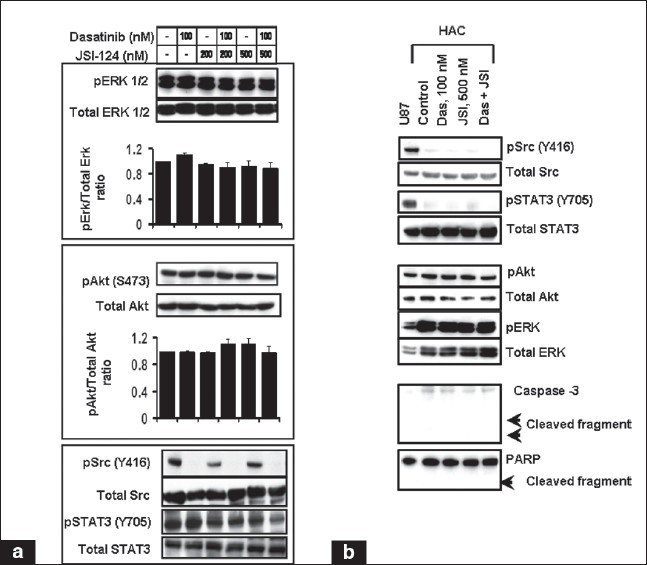
Combination of dasatinib and JSI-124 modulates survival and proapoptotic molecules. U87 (A) or human cerebellar astrocyte (HAC) (B) cells were incubated in the presence of inhibitors at the indicated concentrations for 2 days. Cell lysates were separated by SDS-PAGE and subjected to immunoblot analysis with indicated antibodies. Graphs showing the densitometric analysis of three replicate determinations ± SD are shown. In B, U87 cell lysate was used as a positive control

To determine whether the combined effects of dasatinib and JSI-124 reflected effects on MAPK or Akt signaling pathways, cells were treated with dasatinib, JSI-124 or the combination of both and the lysates were processed for Western blotting with antibodies specific for phospho-Akt, phospho-ERK1/2, phospho-p38, and phospho-JNK. Western blot and densitometric analysis showed that treatment with JSI-124 and dasatinib, alone or in combination, had minimal inhibitory effects on either phospho-Akt or phospho-ERK1/2 [Figure 7a], pJNK or p38 (data not shown). To address whether the synergy between dasatinib and JSI-124 resulted from potentiating effects on Src and STAT3 activation, the level of Src (Y416) and STAT3 (Y705) phosphorylation was examined. As expected, dasatinib abolished Src phosphorylation but produced minimal inhibition of STAT3 phosphorylation [Figure 7a]. However, combination of dasatinib and JSI-124 resulted in additive inhibition of STAT3 [Figure 7a] and cyclin E and upregulation of p21 (data not shown).

We noted that the viability of normal human astrocytes and human cerebellar astrocytes was not significantly affected by Src and STAT3 inhibition or the combination of both [Figure 4f and 5c]. To further address the issue, we evaluated the key signaling molecules in human astrocytes compared to the U87 glioma cell line. As shown in Figure 7b, Src and STAT3 was over-activated in U87 glioma cells as compared to primary astrocytes. Reprobing the blot with total Src and STAT3 shows that variability in protein loading could not account for the observed differences. Human astrocytes showed high levels of pAkt, and pERK. Western blot analysis revealed that there was no significant change in the levels of phosphorylation status of Akt and ERK after inhibitor treatment. We found no induction of cell death in human astrocytes treated with JSI-124 or dasatinib alone or with the combination of both as assessed by caspase 3 and PARP activation. This was consistent with the recent observation by Konnikova *et al*., who have shown that targeting STAT3 by siRNA induced tumor cell death, but did not kill normal astrocytes.[50] Together, these findings demonstrate that Src and STAT3 signaling plays a critical role in glioma cell survival and proliferation and that treatment of cells with dasatinib and JSI-124 results in decreased cell viability and induces apoptosis. In contrast, normal astrocytes showed relatively little or no phosphorylated Src or STAT3 expression. This suggests that Src and STAT3 may be an ideal target for glioma therapy since inhibition of STAT3 signaling induces tumor cell death, but does not kill normal astrocytes.

## DISCUSSION

Src kinase activity has been implicated in promoting key oncogenic mechanisms such as cell proliferation, adhesion, invasion, and resistance to apoptosis.[4,5] In the present study, we have shown that dasatinib, an inhibitor of SFK, interacts synergistically with JSI-124, an agent known to inhibit STAT3/JAK pathways that are involved in glioma growth and survival. Furthermore, depletion of Src and STAT3 by siRNA inhibits cell proliferation and migration, suggesting that Src and STAT3 together play a crucial role in these cellular responses. Dasatinib effectively inhibited phosphorylation of Src as well as downstream signaling molecules and migration of glioma cell lines *in vitro* at physiologically achievable concentrations (≤ 100 nM). These results are consistent with previously published data in sarcoma cell lines.[51] Dasatinib inactivated FAK, suggesting that FAK may be involved, at least in part, in glioma cell migration. Disruption of cytoskeletal architecture upon treatment with this agent resulted in more compact, rounded cells that lacked the extensively branched actin structures normally seen in glioma cells. The effects of dasatinib on migration suggest that Src inhibition may be of therapeutic benefit in inhibiting glioma cell motility.

However, dasatinib had no significant effect on cell proliferation and viability *in vitro* at clinically achievable concentrations. In general, our findings are in agreement with those reported by Finn *et al*.,[23] and Huang *et al*.,[52] in that dasatinib caused a modest growth inhibition at low micromolar concentrations in breast cancer cell lines. The IC50 of ≥ 10 *µ*M for glioma cell lines (100 times that needed to inhibit Src phosphorylation) suggests cytotoxicity may be due to potential off-target effects instead of the direct inhibition of Src kinase activity.

Recent studies have demonstrated that activated STAT3 is over-expressed in human malignant glioma tissues and cell lines.[53,54] JSI-124 is of potential therapeutic interest, both because it directly inhibits the growth of STAT3-active tumors, and because it promotes the differentiation of dendritic cells.[55,56] Because the Src and STAT3 pathways may both be involved in transducing proliferative and survival signaling from dysregulated cell surface receptors, we hypothesized that the combination of dasatinib and JSI-124 might promote glioma toxicity by decreasing the activation status of both Src and STAT3 in parallel, as well as downregulating the levels of other relevant signaling effectors. Our results indicate that interfering with Src and STAT3 signaling by these pharmacological inhibitors resulted in enhanced effects against cell proliferation and migration, as well as potentiation of apoptotic signaling.

We [57,58] and others [46–48] have shown that translocation of AIF and cytochrome *c* from the mitochondria to the nucleus is a critical step for the induction of apoptosis and that this process initiates nuclear condensation,[59] chromatin fragmentation, and cell death.[60] Consistent with these findings, we found translocation of cytochrome *c* and AIF from the mitochondria to the nucleus as early as 1 h after dasatinib and JSI treatment. Although MAPK and Akt signaling does not appear to be affected under the conditions examined in this study, dasatinib and JSI-124 inhibit Src and STAT3, resulting in inactivation of target genes that are responsible for proliferation. It is clear that SFK and STAT3/JAK inhibitors have synergistic effects by promoting cytochrome *c* release and activating several proapoptotic and antiproliferative pathways. In summary, the combination of Src and STAT3 inhibition appears to achieve significant potentiation in diminishing both proliferation and migration in glioma cells. Further work in both animal models and clinical trials is necessary to further validate the potential utility of this combination approach as a therapeutic strategy.

## AUTHOR’S PROFILE

**Dr. Daniel R. Premkumar**, Ph.D., is Research Assistant Professor of Neurological surgery at the University of Pittsburgh. The major research emphasis is directed towards understanding the molecular mechanisms of receptor tyrosine kinase inhibitors and signaling in malignant human glioma cell lines. He is currently examining the efficacy of promising receptor inhibitors for inhibiting glioma proliferation in vitro, using a genotypically diverse panel of malignant glioma cell lines to identify potential genotype-response associations.

**Ms. Naomi Agostino**, B.S. is research technician at the Department of Neurosurgery

**Dr. Esther P. Jane**, Ph.D., is Research Assistant Professor of Neurological surgery at the University of Pittsburgh. Her major research interest is to evaluate strategies for the combination of agents with distinct mechanisms of signaling inhibition to achieve enhanced effects on downstream signaling pathways in order to arrest the growth of glioma cells.

**Mr. Joe Scialabba** is an undergraduate student at the University of Pittsburgh

**Dr. Ian Pollack**, MD, is co-director of the Brain Tumor Program at the University of Pittsburgh Cancer Institute, chief of Pediatric Neurosurgery at Children’s Hospital of Pittsburgh, and Walter Dandy Professor of Neurosurgery at the University of Pittsburgh School of Medicine. Dr. Pollack graduated magna cum laude from Emory University, where he earned a BS degree in chemistry. He received his medical degree from the Johns Hopkins University School of Medicine, then completed a surgical internship and neurosurgical residency at the University of Pittsburgh School of Medicine. Dr. Pollack has published more than 200 papers in refereed journals, numerous book chapters and invited papers, and has edited two books on childhood brain tumors. He is co-editor of the recently published book Principles and Practice of Pediatric Neurosurgery. He is currently a principal investigator on numerous NIH grants focusing on the novel strategies for brain tumor treatment.


